# The pan American health organization: new challenges, new roles, new responsibilities

**Published:** 2013-03-30

**Authors:** Diego A. Bernardini-Zambrini

**Affiliations:** 1 Pan American Health Organization PAHO/WHO. Regional Office for the Americas, World Health Organization, Washington D.C, E-mail:diegobernardiniMD@gmail.com

PAHO is the oldest international public health agency. It was created in 1902 as a mandate from a regional group of countries. Besides currently serving as WHO's Regional Office for the Americas it is the specialized health agency for the Organization of American States (OAS); thus it must render accountability at two levels. PAHO is intimately linked to the development of regional health in the Americas and, therefore, to societal development in the countries from which it is composed. A new Director has been elected this year, Dr. Carissa Etienne, who will guide the institution for at least the next five years.

Thus PAHO is experiencing a period of change, a period of great relevance for defining the architecture, operation and agenda of the organization. The new role of the organization will be formed from the definition of the political action agenda, generating broad consensus on regional and global levels, as well as the public record of the health priorities and actions of the institution. Transforming the needs, the desires into effective public policies and communicating effectively its achievements and aspirations, will not only increase its support but legitimize its technical authority. This can be distilled as capacity for leadership.

One of the short-term debates about the role of this senior public health organization is how it will materialize a universal model of health based on equality in a region of high inequality. This is undoubtedly the greatest challenge. In an era of social determinants of health, of social justice and equity, and of human rights, Universal Health Coverage is a step forward in carrying out these statements and theories using evidence. Other discussions include new approaches to health, new approaches from different areas of knowledge which will enhance the primary health care dimension based on network integrated work and the synergies offered by the private sector and the civil society. It's all a part of the new reality being experienced by PAHO.

For all this, the Organization counts on solid and accepted recognition as consulting agency for best practices and for developing ideas in a world of constant change, with multi-polarities and solutions with multiple partners. These are times where synergies tend to favor interventions through horizontal programs. Therefore, the Organization must confront great strategic and management challenges - both internal and external - produced by the changes at the regional level. It will be necessary to define and create a consensus for the vision of a new role in a scenario where resources are scarce, institutions are involved and coordination for efficiency and effectiveness is the greatest of challenges. Given that in this same region, and particularly in the field of health, multilateral and bilateral institutions, foundations and NGOs, among others have coexisted for a long time.

The global context offers the unique opportunity to be able to assist with making a quantitative and qualitative leap in public health. Today we face a new reality where health is strongly linked to aspects of development. We are embedded in a region where economic growth is leading to a sequence in which there is a greater distribution of income, an expansion of the middle class, an alleviation of poverty, the empowerment of individuals and greater social involvement with the consequent construction of citizenship. This reality allows for a moment of opportunity that has seldom been so conducive. Health is a result of a country's development, but it is also necessary to make it happen. PAHO will require a number of central measures to recover and direct its historical leadership. To achieve this it will require the re-thinking and re-orienting of an organization that has more than 110 years of history, and a seal that inspires adaptation to the current situation.

In a world of constant and rapid change flexibility is necessary. In a region with consolidated democratic governments and with developing broad integrative sub-regional processes, an organization must promote and take active part in this integration. In a highly competitive scenario one should be efficient and effective; invigorating those strategies that have been successful in meeting needs and thinking about those that will enable us to confront the challenges ahead. In a world of economic instability where southsouth cooperation, which has shown itself to be a valid strategy, must consider the countries of the region whose economies will be playing leading roles in the immediate future: Mexico and Brazil, and in the second tier, represented by economies such as Chile, Colombia and Peru, that will permit a greater degree of autonomy.

This should be accompanied by monitoring and evaluation that allows for quantifying how, where, and at what cost has the implementation of programs that compose technical health cooperation been accomplished. The implementation of commitments must be managed effectively.

Today, health has become a dimension of public consciousness that allows room for dialogue, for integration, and for making sense of solidarity, key elements that make for transparency and accountability of an organization like PAHO. As Schumpeter has stated, one must be innovative in the sense of an organizational change that results in quantitative and qualitative aspects, in a greater and better dynamics that permeate a large network of partners and accesses greater resources. In this way, the development of a vision and a clear long-term strategy, defined and appropriate to the current agenda, will mark the continuity and the role of the longest standing public health agency to date.

